# Promotion effect of apical tooth germ cell-conditioned medium on osteoblastic differentiation of periodontal ligament stem cells through regulating miR-146a-5p

**DOI:** 10.1186/s12903-022-02485-8

**Published:** 2022-11-25

**Authors:** Yueqiang Xie, Yaxin Zheng, Liangjiao Chen, Zedong Lan

**Affiliations:** 1 Department of Orthodontics, Stomatological Hospital, Southern Medical University, Guangzhou, 510140 Guangdong China; 2 Department of Orthodontics Division I, Stomatological Hospital of Xiamen Medical College; Xiamen Key Laboratory of Stomatological Disease Diagnosis and Treatment, Xiamen, Fujian China; 3 Department of Orthodontics, Stomatological Hospital of Guangzhou Medical University, Guangzhou, China; 4 Department of Orthodontics, Shenzhen Stomatological Hospital of Southern Medical University, Shenzhen, 518000 Guangdong China

**Keywords:** Osteoblastic differentiation, PDLSCs, miR-146a-5p

## Abstract

**Background:**

MicroRNAs (miRNAs) play an important role in gene regulation that controls stem cells differentiation. Periodontal ligament stem cells (PDLSCs) could differentiate into osteo-/cementoblast-like cells that secretes cementum-like matrix both in vitro and in vivo. Whether miRNAs play key roles in osteoblastic differentiation of PDLSCs triggered by a special microenviroment remains elusive. In this study, we aimed to investigate potential miRNA expression changes in osteoblastic differentiation of PDLSCs by the induction of apical tooth germ cell-conditioned medium (APTG-CM).

**Methods and results:**

First, we analyzed the ability of APTG-CM to osteogenically differentiate PDLSCs. The results exhibited an enhanced mineralization ability, higher ALP activity and increased expression of osteogenic genes in APTG-CM-induced PDLSCs. Second, we used miRNA sequencing to analyze the miRNA expression profile of PDLSCs derived from three donors under 21-day induction or non-induction of APTG-CM. MiR-146a-5p was found to be up-regulated miRNA in induced PDLSCs and validated by RT-qPCR. Third, we used lentivirus-up/down system to verify the role of miR-146a-5p in the regulation of osteoblastic differentiation of PDLSCs.

**Conclusions:**

In conclusion, our results demonstrated that miR-146a-5p was involved in the promotion effect of APTG-CM on osteoblastic differentiation of PDLSCs, and suggested that miR-146a-5p might be a novel way in deciding the direction of PDLSCs differentiation.

## Introduction

**Fig. 1:**
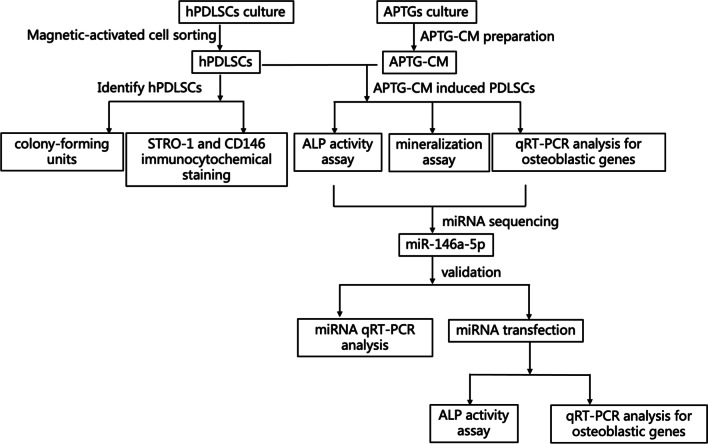
An overview of the study procedures and design

The periodontal ligament (PDL) is a soft connective tissue embedded between the cementum and the inner wall of the alveolar bone socket. PDL contains heterogeneous cells which are a population of multipotential cells that can self-renewing and differentiate into bone-forming cells, cementum-forming cells or other cell lines [1]. These multipotent postnatal stem cells are called periodontal ligament stem cells (PDLSCs) [2]. They have some features, involving the capacity to form mineralized nodules in vitro, response to bone-inductive factors, and the expression of the bone-associated markers alkaline phosphatase and bone sialoprotein, the so-called osteoblast-like properties [3, 4]. Previous studies reported that PDLSCs could differentiate into osteo-/cementoblast-like cells which secreted cementum/PDL-like matrix both in vitro and in vivo as triggered appropriately [5, 6]. Tooth development relies on reciprocal and reiterated molecular signal activity between epithelium and mesenchyme to control morphogenesis and development. The reciprocal and sequential natures of these inductions has been found to be the basis for advancing differentiation of dental tissues [7, 8]. Tooth germ cell-conditioned medium (TGC-CM) could transform dental pulp stem cells and generated a regular-shaped dentin-pulp complex containing distinct dentinal tubules and predentin in vivo [9]. Apical tooth germ cells (APTGs) consisting of both epithelial and mesenchymal cells could provide a specific microenvironment for tooth root regeneration. Besides, apical tooth germ cell conditioned medium (APTG-CM) was demonstrated to provide a cementogenic microenvironment and induced the differentiation of PDLSCs toward cementoblastic lineage [10]. However, the effect of APTG-CM on the osteoblastic differentiation and molecular interaction among PDLSCs is not well known. MicroRNAs (miRNAs) are a kind of 20- to 22- nucleotide-long small non-protein-coding RNAs that negatively regulate gene expression at the post-trancriptional level [11]. Many microRNAs play functions in stem cells differentiation [12]. It has been reported that microRNAs played a crucial role in the osteogenic differentiation of human bone marrow mesenchymal stem cells [13]. It has also been reported that miR-146a promoted the differentiation of PDL cells [14]. In this study, we investigated the effect of induction of APTG-CM on the osteogenic differentiation of PDLSCs and the potential role of regulation of miR-146a during osteogenesis of PDLSCs (Fig. 1).

## Methods and materials

### Immunomagnetic sorting of PDLSCs and PDLSCs identification

PDLs were isolated from normal premolars extracted from four patients (aged from 13–14 years, 2 for males and 2 for females) who sought orthodontic treatment. Written informed consent were provided by all patients and their guardians, and ethical approval had been obtained from the Ethics Committee of Stomatological Hospital of Guangzhou Medica University. PDLs were seperated from the middle third of the roots gently and digested in 3 mg/ml Type I Collagenase as well as 4 mg/ml Dispase for 1 h at 37 °C. Single-cell suspensions were seeded into 25 cm^2^ flask with a-MEM supplemented with 10% fetal calf serum, 100 μmol/l ascorbic acid 2-phosphate, 2 mmol/l glutamine, 100U/ml penicillin, and 100 μg/ml streptomycin. The cultures were incubated in a humidified atmosphere of 5% carbon dioxide and 95% air at 37 °C. To isolate putative periodontal ligament stem cells, subconfluent secondary cultures of periodontal ligament cells were separated by using immunomagnetic cell sorting technique as previously reported [15]. According to the manufacturer’s instructions, single-cell suspensions of PDLCs were incubated with mouse anti-human STRO-1 IgM primary antibody (Life technologies, USA) on a rotator for 1 h at 4 °C, as STRO-1 was the best-known mesenchymal stem cell marker. Wash the cells by using phosphate buffered saline containing 0.1% bovine serum albumin (BSA) and 2 mM EDTA, and resuspended with Dynabeads rat anti-mouse IgM for 45 min on a rotator at 4 °C. All bead-positive cells were isolated with Dynamag 15 magnetic particle concentrator and cultured in Nutristem MSC Basal Medium supplemented with Nutristem MSC XF Supplement Mix MSC (Biological Industries, Israel), while the bead-negative cells were incubated in a-MEM medium. To assess colony-forming efficiency, after 12 days, subconfluent cultures (second passage) of MSC positive and negative cells were fixed with 70% ethanol and then stained with 0.1% Crystal Violet. Aggregates of over 50 cells were counted as a colony under microscopic observation. To analyze the expression of relative markers of PDLSCs, subconfluent secondary cultures of bead-positive and bead-negative cells were seeded at 1 × 10^3^ cells per well on a eight-chambered slide. All the cells were fixed in 4% paraformaldehyde for 20 min at room temperature and blocked with phosphate buffered saline containing 1% BSA as well as 0.3%Triton-100X for 30 min at room temperature. Then the samples were incubated with primary antibodies at 4 °C overnight. The primary antibodies were as follows, mouse anti-human STRO-1 IgM, mouse anti-human CD146, mouse anti-human vimentin, mouse anti-human cytokeratin (Life Technologies, USA). The cells were incubated with donkey anti-mouse secondary antibodies of IgG-FITC (Jackson, USA) for 45 min at 37 °C subsequently. Cell nuclei were stained with 0.25 μg/l Hoechst 33342 (Life Technologies, USA) for 5 min at room temperature. Cell staining was evaluated using the fluorescence microscope (Leica CTR6000, Germany).

### APTG-CM preparation

**Fig. 2:**
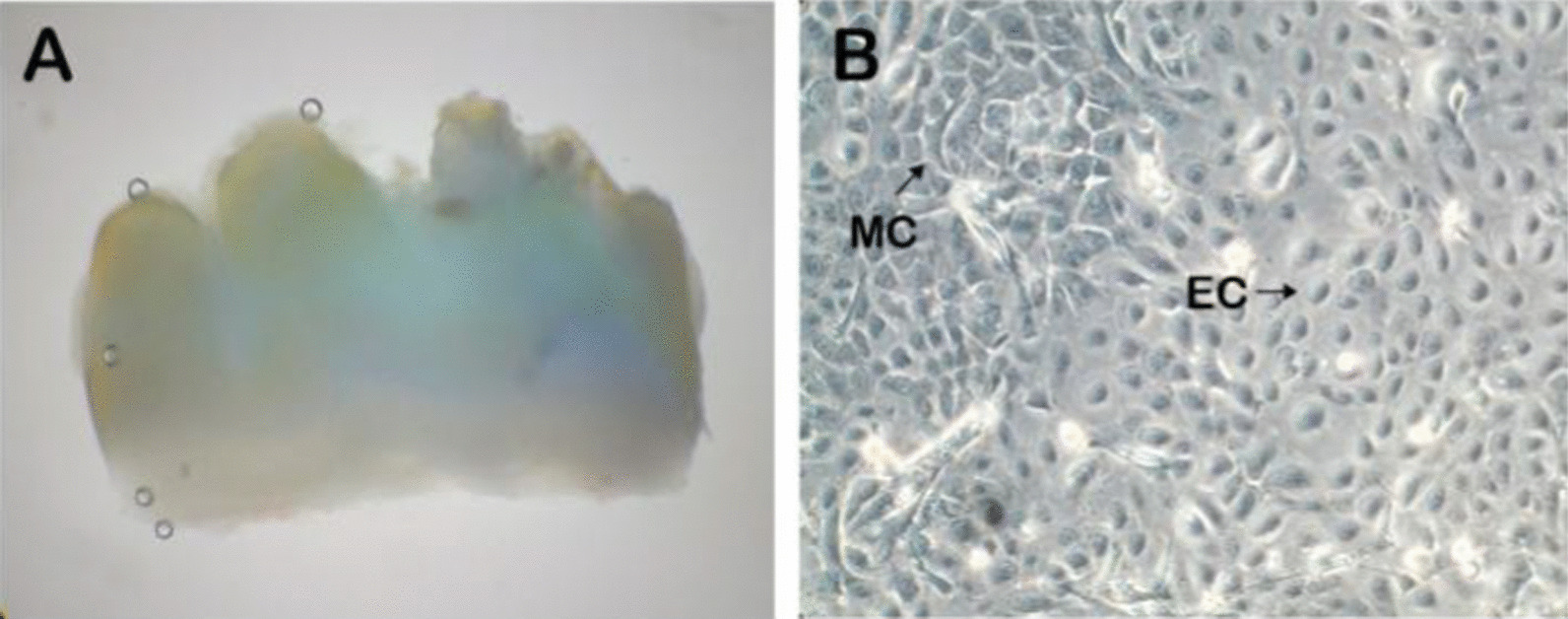
Isolation and culture of APTGs. A mandibular developing first molar germ of a 8-day-old Sprauge-Dawley rat (**A**). The cultured epithelial and mesenchymal apical tooth germ-derived stem cells. EC and MC refer to epithelial and mesenchymal cells respectively (**B**)

APTG-CM was made as previously reported [2]. The use of Sprague-Dawley rats (Laboratory Animal Center of Sun Yat-sen University) and the experimental protocols were approved by Animal Care Committee of Guangdong Province. Eight-day postnatal Sprague-Dawley rats were killed by cervical dislocation. Twenty molar germs were removed from mandibles under a stereomicroscope, and Hertwigs epithelial root sheath (HERS) associated with apical mesenchyme was carefully dissected from the apical root of molar germs (Fig. 2A). The apical portions of tooth germ were minced into < 1 mm^3^, and digested in 3 mg/ml Type I Collagenase as well as 4 mg/ml Dispase for 1 h at 37 °C. Single-cell suspensions were seeded into 75cm^2^ flask at 1 × 10^5^ cells/ml with a-MEM supplemented with 10% fetal calf serum, 100 μmol/l ascorbic acid 2-phosphate, 2 mmol/l glutamine, 100 U/ml penicillin, and 100 μg/ml streptomycin. The cultures were incubated in a humidified atmosphere of 5% carbon dioxide and 95% air at 37 °C. The culture medium of primary apical tooth germ cells containing both epithelial and mesenchymal cells was changed every 48 h until full confluence (Fig. 2B). The medium was collected and centrifuged at 2000*g* for 15 min 3 days after the last medium changed. Then the supernatants, which were mixed with an equal volume of fresh a-MEM medium, were used as APTG-CM and stored at − 20 °C for PDLSCs culture.

### Alkaline phosphatase (ALP) activity assay

PDLSCs were inoculated into 12-well culture dish (2 × 10^3^ cells/well) and cultured with or without APTG-CM for 4, 8, 12 and 16 days. The ALP activity was detected with an assay kit (Jiancheng Co, Nanjing, China) according to the manufacture’s instructions. Briefly, cells were harvested using protein lysis buffer containing 50 mM Tris–HCl, 150 mM NaCl as well as 1%NP-40. Then, cells were incubated with the applied phenol standard liquid, buffer and the substrate solution for 15 min at 37 °C. After adding the color-substrate solution, all the experiments were performed in triplicate in three different experiments using the same cells and tested at 520 nm wavelength by a enzyme micro-plate reader.

### Mineralization assay

The mineralization assay in vitro were operated as previous described [16]. The PDLSCs (1 × 10^5^/well) were cultured to confluence in Nutristem MSC Basal Medium supplemented with Nutristem MSC XF Supplement Mix MSC in six-well cuture dishes. Then, the cells were cultured in two different medium conditions. The induced cells were grown in APTG-CM, while the non-induced cells were cultured in Nutristem MSC Basal Medium. After 14 and 21 days, the cells were fixed with 4% paraformaldehyde for 20 min, and stained with 2% Alizarin Red Solution for 30 min at room temperature. The mineralized nodules were imaged by microscope (Zeiss, Germany). Then added 1.5 ml 0.5 N HCl into each well for 30 min and collected the supernatent. The absorbance of each supernatent was measured by spectrophotometer at 405 nm wavelength (Thermo, USA).

### Real time q-PCR analysis for osteoblastic gene

Total RNAs were isolated from 21-day APTG-CM-induced and non-induced PDLSCs derived from four individual donors (Sample A, B, C and D) using mirVanaTM miRNA isolation kit (Life Technologies, USA) according to the manufacture's instructions. The quantity and purity of the total RNA were verified by spectrophotometer. Reverse transcription reaction was performed using reverse transcriptase M-MLV (Takara, Japan), and mRNA expression levels were quantified using Light Cycler 480 SYBR Green I Master (Takara, Japan). The primers of osteoblastic target genes were listed as in Table 1. Relative quantification presented qPCR data of target genes relying on internal control gene GAPDH as reference. PCR conditions of Light Cycler 480 (Roche, Switzerland) were as follows: pre-denaturation at 95 °C for 5 min, followed by 40 cycles of denaturation at 95 °C 15 s, primer annealing at 56 °C for 15 s, and extension at 72 °C for 25 s.

**Table 1:** Primers used for Real-time RT-PCR are listed

Name	Primer
ALP	5ʹ-TTCAAACCGAGATACAAGCACT-3ʹ
	5ʹ-GGGCCAGACCAAAGATAGAG-3ʹ
BSP	5ʹ-GAACCACTTCCCCACCTTTTG-3ʹ
	5ʹ-ATTCTGACCATCATAGCCATCT-3ʹ
PLAP-1	5ʹ-CGATACAAGAACTACAAAGGCT-3ʹ
	5ʹ-TGCATTTCCCAGTATTTCACCG-3ʹ
GAPDH	5ʹ-AGGTCGGAGTCAACGGATTTG-3ʹ
	5ʹ-AGGCTGTTGTCATACTTCTCAT-3ʹ

### Illumina miRNA sequencing

Total RNAs of 21-day APTG-CM-induced and non-induced PDLSCs derived from three individuals (Sample A, B and C) were isolated by using mirVana™ miRNA isolation kit (Life Technologies, USA) and sequenced for analysis, which were sent to Shanghai Biotechnology Corporation. Briefly, 3' and 5' adapters were sequentially ligated to small RNA, and single-stranded RNA molecules with adapters at both ends were amplified for 11 cycles using a common primer and a primer index. Then the cDNA was purified in 6% Novex TBE PAGE Gel, and the gel slice corresponding to the DNA size was excised and eluted in 1 × gel elution buffer. DNA High Sensitivity Chip of Aglient 2100 (Aglient Technologie, USA) was used in the quality control. After that, the purified cDNA was used for cluster generation and sequencing analysis using Agilent Technologies 2100 Bioanalyzer ( Agilent Technologie, USA).

### miRNA qRT-PCR analysis

Total RNAs of 21-day APTG-CM-induced and non-induced PDLSCs derived from Sample D were isolated using mirVanaTM miRNA isolation kit (Life Technologies, USA). The designed miR-146a-5p-specific primer and U6B internal control primers were listed in Table 2, Quantitative RT-PCRs for miRNA were performed with SYBRe PrimeScript miRNA RT-PCR Kit (Takara, Japan). The PCR reaction were conducted utilizing LightCycler 480 (Roche, Switzerland) as following conditions: 1 cycle of pre-denaturation at 95 °C for 30 s, followed by 40 cycles of: 5 s at 95 °C and 20 s at 60 °C.

**Table 2:** Primers used for qRT-PCR

Name		Primer
U6B	F	CTCGCTTCGGCAGCACA
U6B	R	AACGCTTCACGAATTTGCGT
miR-146a-5p		UGAGAACUGAAUUCCAUGGGUU

### miRNA transfection

In order to validate the regulatory effects of miR-146a-5p on osteoblast differentiation of PDLSCs in vitro, recombinant lentivirus miR-146-5p (Lenti-miR-146-5p) and lentivirus anti-miR-146-5p (Lenti-anti-miR-146-5p), along with lentivirus miR negative control (Lenti-miR-NC) and lentivirus anti-miR negative control (Lenti-anti-miR-NC), designed and provided by Genechem lnc. (Shanghai, China, http://www.genechem.com.cn), were used to regulate miR-146a-5p levels in PDLSCs. After the cells reached about 30 mlnc./" l lowotathey were transfected with lentivirus at the desired multiplicity of infection (MOI = 1000) with enhanced infection solution (ENI.S) according to the manufacturer’s protocol. Stably transfected cells were selected with 2 μg/ml puromycin (Sigma, German). The expression of stable transformants was observed under fluorescence microscopes and identified by qRT-PCR.

### Predicted miRNAs targets

A combination of two online softwares, miRWalk (http://mirwalk.umm.uni-heidelberg.de/) and TargetScan (https://www.targetscan.org/cgi-bin/targetscan/hsa-miR-146a-5p+), was used to predict the potential hsa-miR-146a-5p target genes. We selected the identical target genes indicated in both softwares and then did bioinformatics analyses to determine associations between hsa-miR-146a-5p and target genes by PubMed (http://www.ncbi.nlm.nih.gov/pubmed/).

## Statistical analysis

Statistical significance was assessed by Student's *t-*test. The *P* values of less than 0.05 were considered significant.

## Results

### Isolation of PDLSCs

**Fig. 3:**
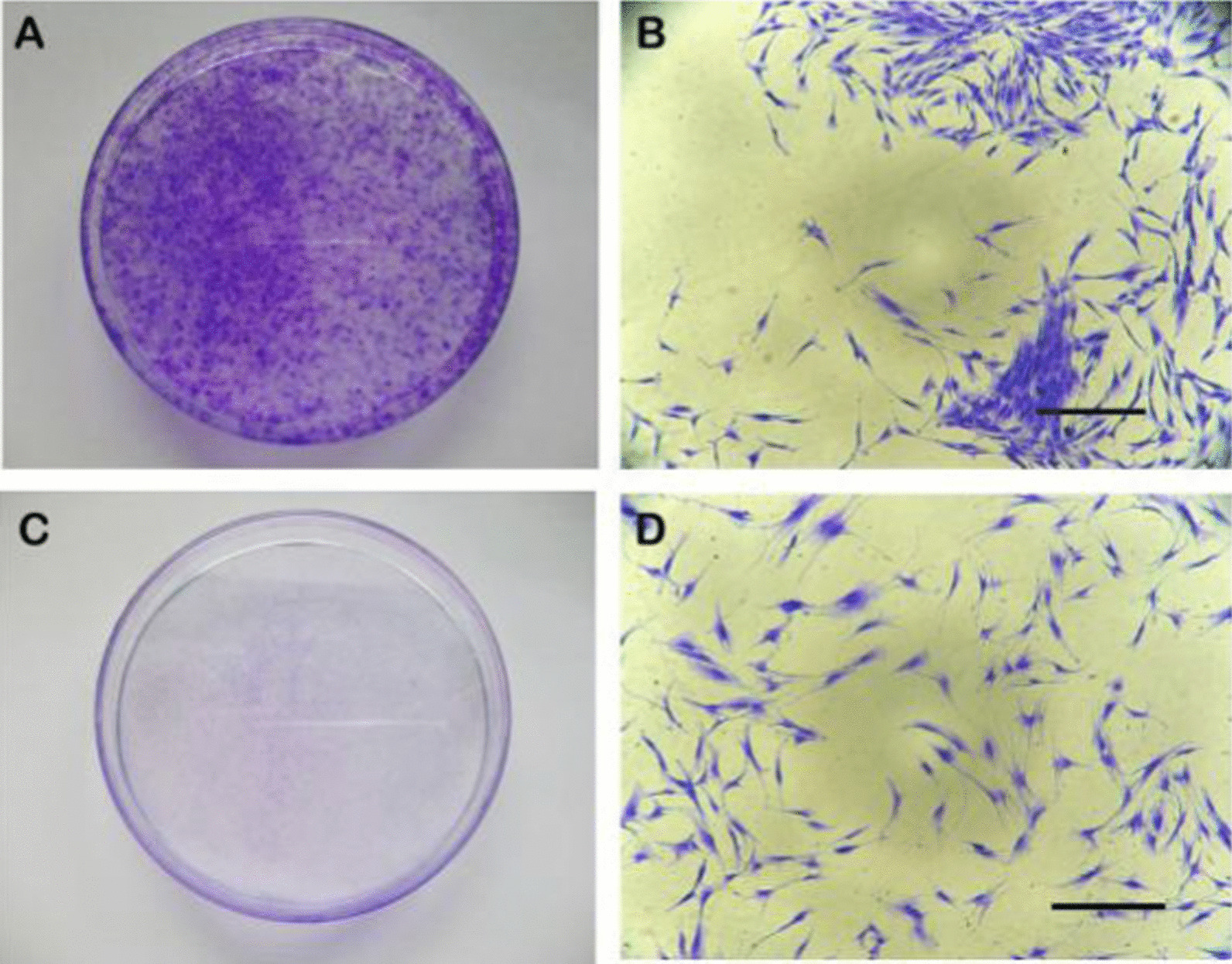
Characteristics of human PDLSCs. MACS positive cells were stained with 0.1% crystal violet and showed several colony-forming units exhibiting typical PDLSCs characteristics (**A**). Aggregates containing more than 50 cells were counted as a colony unit (**B**). MACS negative cells were stained with 0.1% crystal violet and showed no colony-forming unit (**C**, **D**). Scale bar: 400 μm (**B**, **D**)

In this study, PDLSCs were isolated and purified by magnetic-activated cell sorting (MACS). On the twelfth day, the cultures were stained with 0.1% crystal violet, and the numbers of colonies were statistically evaluated. The isolated PDLSCs were capable of forming adherent colonies, and majority of the cells retained their fibroblastic spindle shape (Fig. 3A–B). Aggregates containing more than 50 cells were counted as colonies under the microscope, and several colony-forming units exhibiting typical PDLSCs characteristics were observed. While MACS negative cells were stained with 0.1% crystal violet also and showed no colony-forming unit (Fig. 3C, D).

### Immunocytochemical staining of PDLSCs

**Fig. 4:**
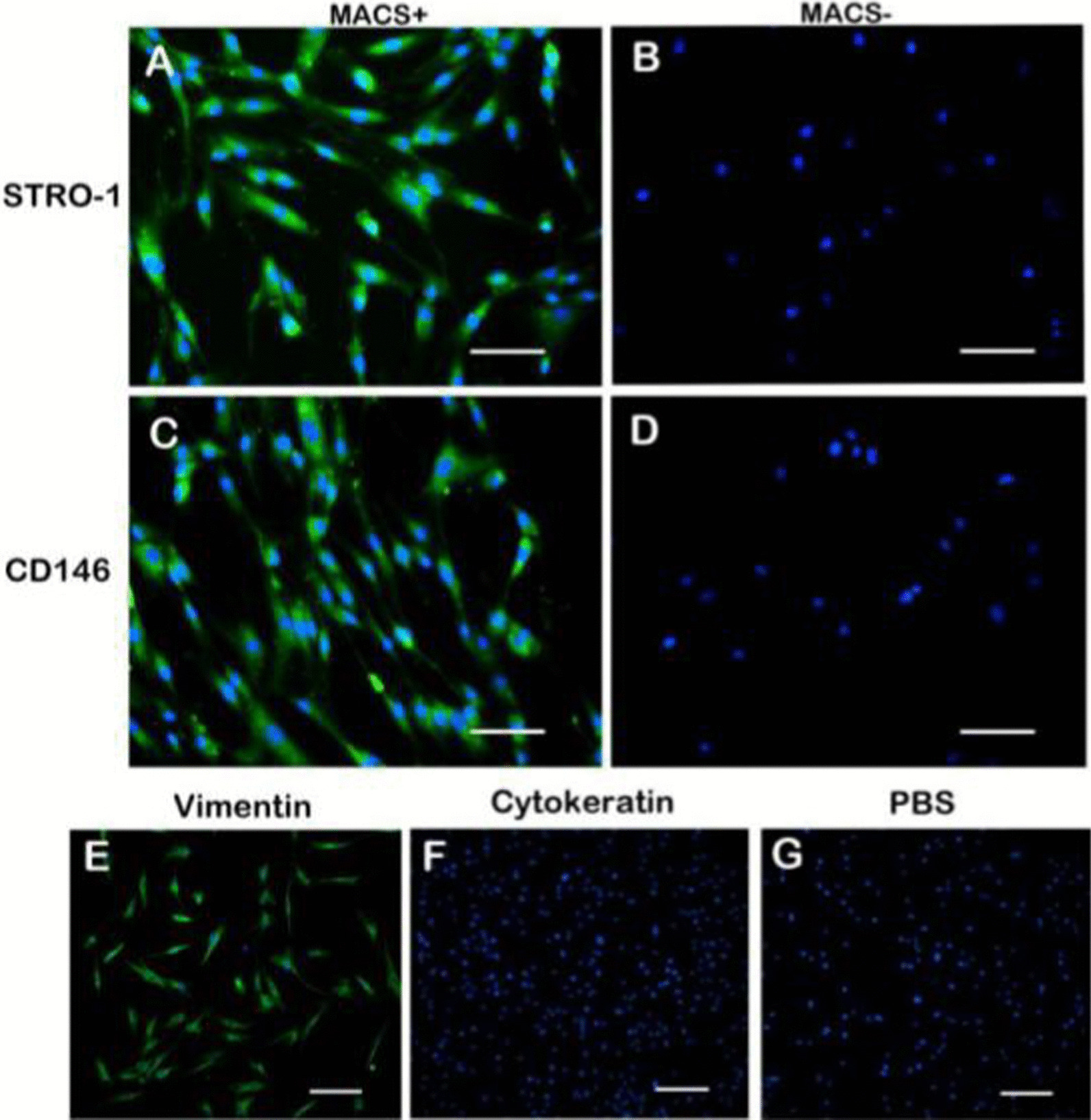
Immunocytochemical staining of PDLSCs. The MACS positive cells showed the expression of STRO-1 (**A**) and CD146 (**C**) by immunocytochemical fluorescent staining, whereas the negative cells didn’t show the expression of STRO-1 (**B**) and CD146 (**D**). PDLSCs could express mesenchymal cell marker vimentin (**E**) but no epithelial cell marker cytokeratin (**F**). Scale bar; 50 μm (**A**–**D**), 100 μm (**E**–**G**)

Immunocytochemical staining was carried out to further identify the PDLSCs. The results showed that all periodontal ligament stem cells could express vimentin but no cytokeratin, which indicated that all cells were isolated from mesenchymal cells without the involvement of epithelial cells (Fig. 4E–G). PDLSCs were fluorescently labeled to confirm the expression of STRO-1 and CD146 using immunocytochemical staining when isolating and purifying cells by MACS. The results showed that the MACS positive cells were fluorescent, whereas the negative cells were free of fluorescence (Fig. 4A–D).

### ALP activity and mineralization formation of APTG-CM-induced PDLSCs

**Fig. 5:**
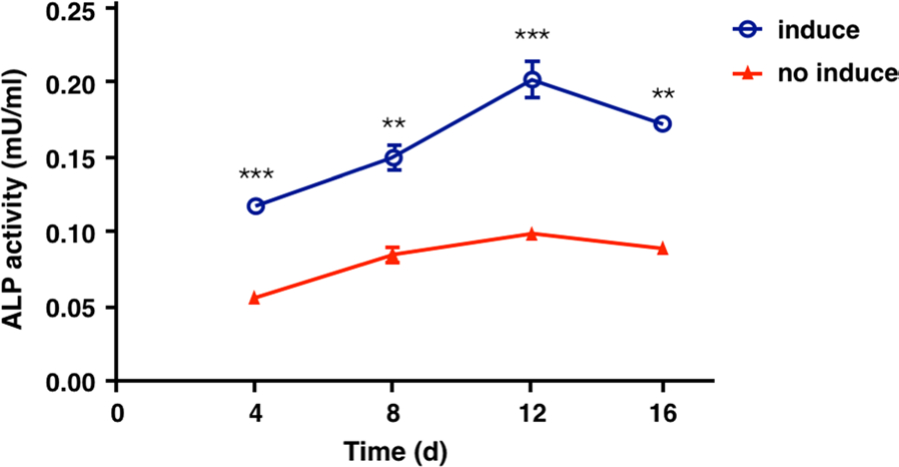
ALP activity of APTG-CM-induced and non-induced PDLSCs. ALP activities of PDLSCs increased gradually during 4–16 day period and were significantly different between induced PDLSCs and non-induced PDLSCs. Data were as mean ± S.D. (n = 3). **P < 0.01; ***P < 0.001

**Fig. 6:**
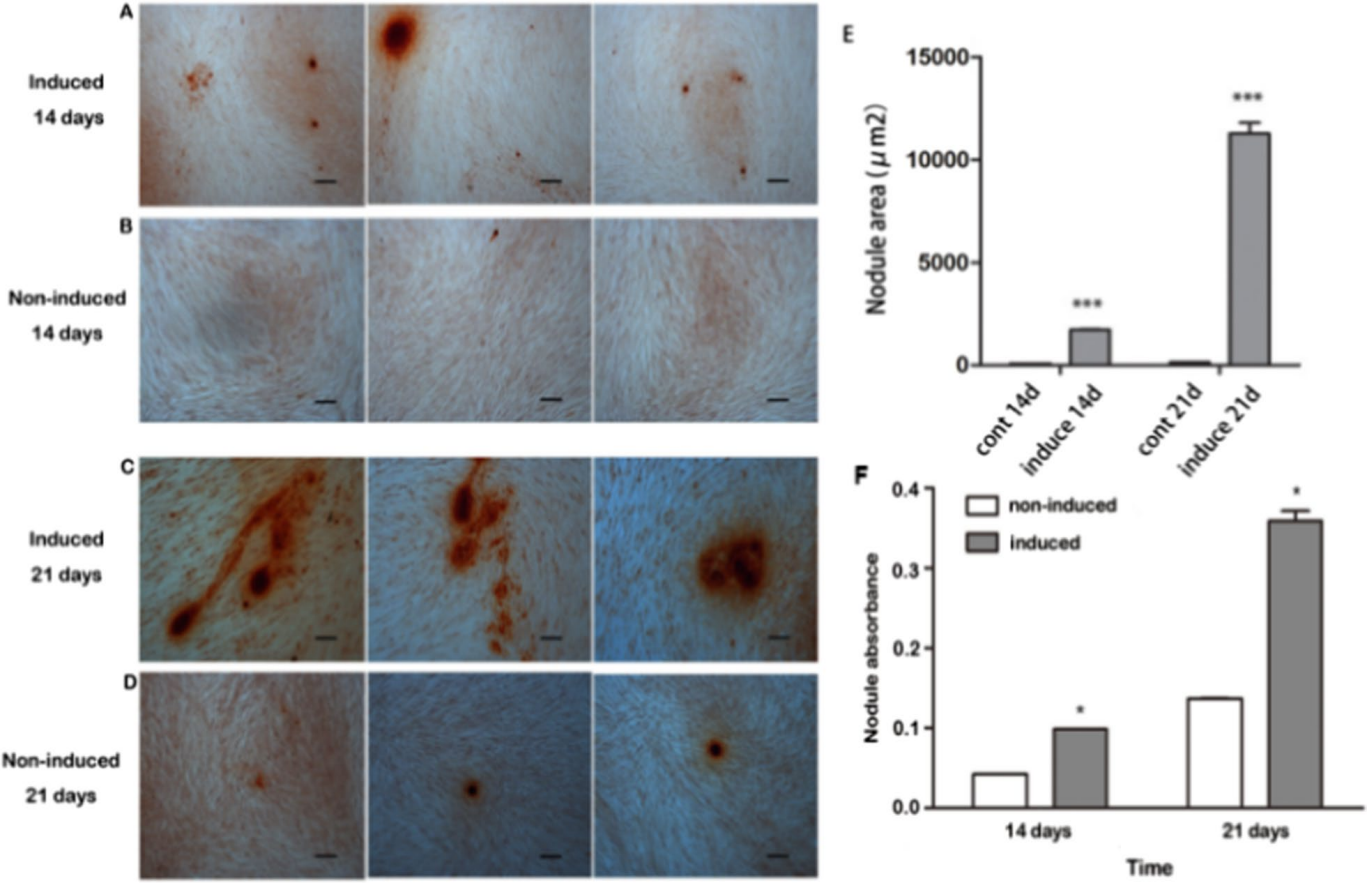
Mineralization formation investigated by Alizerin Red Solution staining. PDLSCs cultured in complete MSC Nutristem medium supplied with 2 mmol/l β-glycerophoshate for a period of 2 (**A**) and 3 weeks (**C**) showed few mineralized nodules. PDLSCs co-cultured with APTG-CM for a period of 2 (**B**) and 3 weeks (**D**) showed the abundance of nodules. Nodule areas were significantly higher in APTG-CM-induced PDLSCs than non-induced PDLSCs (**E**). *P < 0.05; ** *P* < 0.01; ***P < 0.001. Scale bar: 100 μm (**A**–**D**)

APTG-CM-induced PDLSCs showed significantly higher ALP activity than the non-induced PDLSCs after 4, 8, 12, 16 days in culture (Fig. 5). The ALP activity from both groups increased gradually during 0–12-day period and reached the highest level of the ALP activity, and then gradually declined during the 12–16-day period. After 14 and 21 days in culture, APTG-CM-induced and non-induced PDLSCs were stained with 2% Alizarin Red Solution (Sigma, USA), which showed calcium deposits in the induced PDLSCs but few calcium deposits in the non-induced PDLSCs (Fig. 6). The statistical analysis showed that results of absorbance values were the same as above mentioned ones. Taken together, our results indicated that APTG-CM could promote the ALP activity of PDLSCs and enhance mineralization formation.

### Expression levels of osteoblastic genes

**Fig. 7:**
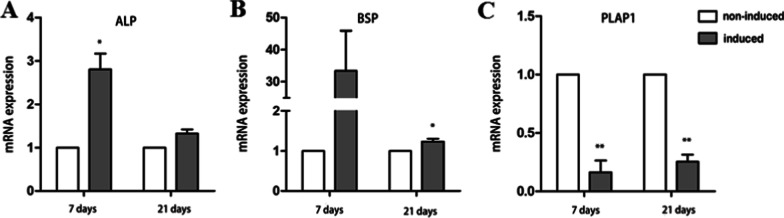
Real-time PCR analysis of osteoblast differentiation-related gene expression. The mRNA expression of ALP (**A**) and BSP (**B**) was significantly increased after 7 days in co-culture with APTG-CM, while the mRNA expression of PLAP1 (**C**) was significantly decreased after 7 days in co-culture with APTG-CM. Data were as mean ± S.D. (n = 3). **P < 0.01; ***P < 0.001

To evaluate a differentiated and mineralizing tissue type, quantitative real-time PCR was carried out at 7 and 21 days in culture of PDLSCs. Real-time PCR results showed that the ALP expression level of APTG-CM-induced PDLSCs were significantly higher than non-induced cells after a period of 7-day, but there was no significant difference between two groups after a period of 21-day (Fig. 7A). The expression of Bone Sialoprotein (BSP), which was the essential marker of mineralized tissue formation and osteogenesis, increased remarkably after 7 and 21 days in co-culture with APTG-CM (Fig. 7B). Besides, the crucial marker Periodontal Ligament-Associated Protein-1 (PLAP-1) of PDL cells, which was proved to be a negative regulator of periodontal ligament mineralization, presented a significant reduction of expression after 7 and 21 days in co-culture with APTG-CM (Fig. 7C).

### Expression profile analysis of microRNAs

**Fig. 8:**
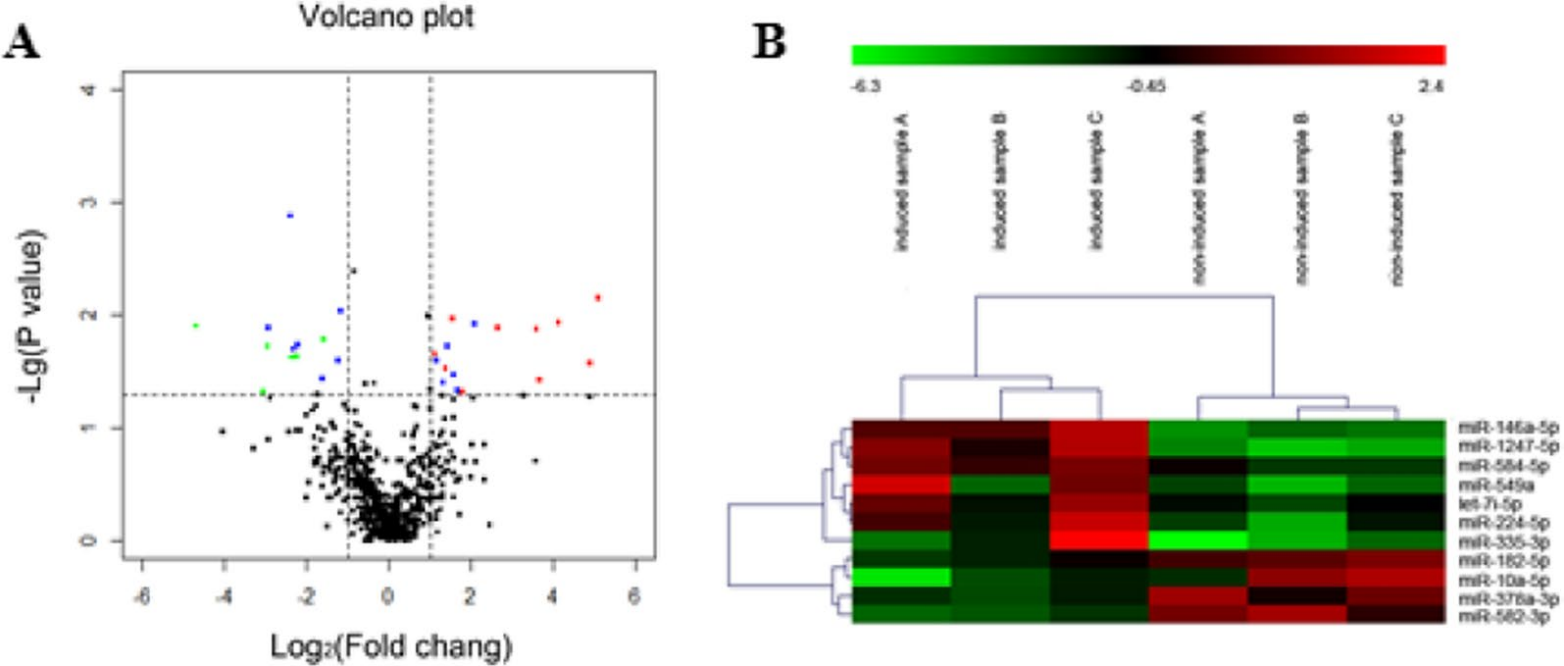
Expression profile analysis of microRNAs. The microRNAs expression profile of APTG-CM-induced and non-induced PDLSCs at 21 days of culture was analyzed by microRNAs sequencing (A is Volcano plot and B is heat map). Graphs displayed the Log2 fold chang (induced versus non-induced). Red dots represented significantly up-regulated microRNAs; green dots represented down-regulated microRNAs; blue dots were counts microRNAs with exact matched < 5

To evaluate expression profile of microRNAs during the differentiation process of PDLSCs, we compared the expression of 1227 known miRNAs between APTG-CM-induced and non-induced cells, and verified that the expression of microRNAs was differentially regulated. After statistical procedures and condition screening (Student’s *t* test. *P* < 0.05, average Log2 (fold change) > 1 and counts of exact matched miRNAs > 5), we found 7 up-regulated microRNAs and 4 down-regulated microRNAs in PDLSCs co-cultured with APTG-CM (Table 3, Fig. 8). We used small RNA sequencing to identify putative novel microRNAs that were never reported before among any species, and found that there were 5 mature microRNA-selective sequences which could be readily tested from sample A, B and C. Five microRNA sequences were GGGGATGTAGCTCAGTGGTAGA, TGCAAAAATAATTGTGGTTTTG, GCTTGACTAGCTTGCTGTTT, AGGATGGCCGAGTGGTCTAAG and GCATTGGTGGTTCAGTGGTAG. They might be a kind of specific microRNAs which played a crucial role in regulating cell differentiation of PDLSCs. Then, we sought to predict target genes and found that miR-146a-5p was involved in the regulation of mineralization.

**Table 3:** Differentially expressed microRNAs (student *t*-test)

microRNA	Mean log (fold change)	*P* value
miR-1247-5p	5.062395619	0.026890418
miR-146a-5p	4.087799757	0.037931894
miR-335-3p	3.91309911	0.004134101
miR-549a	3.10425452	0.023287718
miR-224-5p	2.768368535	0.0108813
miR-584-5p	1.902453044	0.018774097
let-7i-5p	1.456603377	0.007094014
miR-182-5p	− 1.690468832	0.013192304
miR-378a-3p	− 2.180100201	0.01663069
miR-582-3p	− 3.043195747	0.047480095
miR-10a-5p	− 3.398687753	0.01183256

### Hsa-miR-146a-5p expression level by by qRT-PCR

**Fig. 9:**
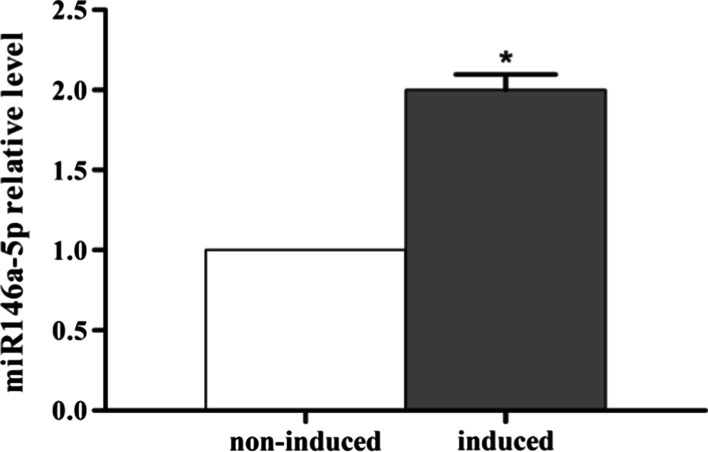
Hsa-miR-146a-5p expression level by qRT-PCR. The expression of hsa-miR-146a-5p was significantly up-regulated in APTG-CM-induced PDLCs versus non-induced PDLCs from sample D. Data were as mean ± S.D. (n = 3). *P < 0.05

To validate the expression of hsa-miR-146a-5p, we used quantitative real-time PCR to assess the expression level of hsa-miR-146a-5p in sample D. The expression of hsa-miR-146a-5p was compared between APTG-CM-induced and non-induced PDLCs of sample D and significantly up-regulated in APTG-CM-induced PDLCs (Fig. 9), which showed the same results as the miRNA sequencing ones from sample A, B, and C.

### Hsa-miR-146a-5p involvement in the regulation of APTG-CM-induced osteogenic differentiation of PDLSCs

**Fig. 10:**
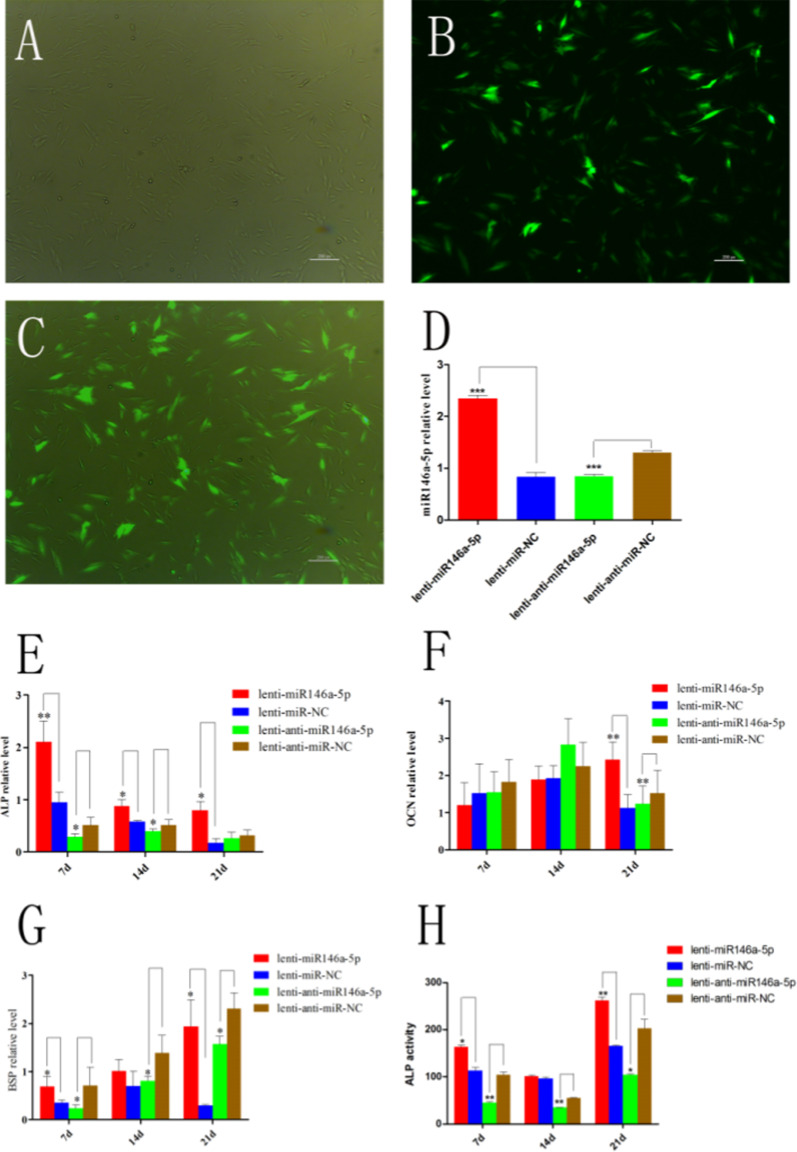
Hsa-miR-146a-5p involvement in the regulation of APTG-CM-induced osteogenic differentiation of PDLSCs. PDLSCs transduced with recombinant lentivirus miR-146-5p were examined by phase contrast microscopy (**A**), fluorescent microscopy (**B**) and merged images (**C**). MiR-146a-5p expression was validated by qRT-PCR, and the results showed that the expression of miR-146a-5p in cells was significantly increased with Lenti -miR-146-5p transduction and decreased with lentivirus anti-miR-146-5p transduction. The expression of OCN, ALP and BSP were detected by RT-qPCR. The results showed that the expression of ALP and BSP was increased in cells transduced with Lenti-miR-126 after 7, 14, 21 days in culture. The results also showed the expression of BSP was decreased in cells transduced with Lenti-anti-miR-126 after 7, 14, 21 days in culture, and the expression of ALP was decreased after 7 days in culture. The expression of OCN was increased and decreased in cells transduced with Lenti-miR-146-5p and Lenti-anti-miR-146-5p after 21 days in culture, respectively (**E**–**G**). Lenti-miR-146-5p-treated groups showed higher ALP activity than control-treated groups, while Lenti-anti-miR-146-5p-treated groups showed lower ALP activity than control-treated groups (**H**). Data were as mean control-treated *P < 0.05; **P < 0.01; ***P < 0.001

To investigate the potential pathways regulated by hsa-miR-146a-5p in APTG-CM-induced osteogenic differentiation of PDLSCs, PDLSCs were transduced with recombinant lentivirus miR-146-5p (Lenti-miR-146-5p), lentivirus anti-miR-146-5p (Lenti-anti-miR-146-5p), lentivirus miR negative control (Lenti-miR-NC) and lentivirus anti-miR negative control (Lenti-anti-miR-NC), respectively (Fig. 10A–C). The relative expression level of miR-146a-5p was detected by qRT-PCR in these four groups. The results showed that the expression of miR-146a-5p was increased in cells transduced with Lenti-miR-146, while the expression was decreased in cells transduced with Lenti-anti-miR-146 (Fig. 10D). Then, we detected the expression level of OCN, ALP and BSP by RT-qPCR in APTG-CM-induced PDLSCs. The results showed that the expression of ALP and BSP was increased in cells transduced with Lenti-miR-146 after 7, 14, 21 days in culture. The results also showed the expression of BSP was decreased in cells transduced with Lenti-anti-miR-146 after 7, 14, 21 days in culture, and the expression of ALP was decreased after 7 days in culture. The expression of OCN was increased and decreased in cells transduced with Lenti-miR-146-5p and Lenti-anti-miR-146-5p after 21 days in culture, respectively (Fig. 10E–G). The results of detection of ALP activity showed that the ALP activity was higher in Lenti-miR-146-5p-treated groups than control-treated groups, while the ALP activity was lower in Lenti-anti-miR-146-5p-treated groups than control-treated groups (Fig. 10H).

### Prediction analysis of hsa-miR-146a-5p target genes

To investigate the potential role of has-miR-146a-5p in maintaining stemness and osteoblastic differentiation, combined bioinformatics analyses were performed in the prediction of miR-146a-5p target genes. Several miR-146a-5p potential target genes were predicted by analyses of computational miRNA target prediction—a combination of miRWalk and miRecord databases. The results were displayed as follows: CXCR4, CCNA2, PA2G4, FADD, CFH, BRCA1, STAT1 and TBP.

## Discussion

PDLSCs exhibit ability of self-renew and show the potentiality to differentiate into ipocytes, osteo/cementoblast-like cells and collagen-forming cells. However, differentiation of the stem cells may be triggered by specific signals provided by the local environment [17]. During tooth development, after the completion of crown formation, the apical mesenchyme develops the periodontium as well as the inner and outer enamel epithelia fuse under the level of the crown cervical margin to produce a bilayered epithelial sheath termed Hertwig's epithelial root sheath (HERS).It has been reported that epithelial–mesenchymal interactions which take place between Hertwig’s sepithelial root sheath and underlying root mesenchyme seem to play an important role in root/periodontal tissue development [18]. Primary APTGs were heterogeneous, containing both cobblestone-like epithelial and spindle-shaped mesenchymal cell types, which provide multiple molecular signals or growth factors necessary for PDLSCs proliferation and differentiation. In this study, primary cultures of human PDLSCs from 4 individual donors was established and their osteo-differentiation potential were confirmed by co-culture with APTG-CM. And then we determined Osteogenic differentiation by mineralization in vitro, osteoblast marker gene expression, and alkaline phosphatase activity. And it was evidenced that APTG-CM-induced PDLSCs exhibited significantly increased calcified nodule formation and enhanced osteogenic gene expression and high ALP activity. The expression of osteogenic differentiation marker genes ALP and BSP was significantly up-regulated, and the expression of PLAP1, which is a negative regulator of periodontal ligament mineralization, was remarkably down-regulated. In contrast, non-induced PDLSCs exhibited low ALP activity and reduced mineralization formation. The results mentioned above showed that PDLSCs had the potentiality to differentiate into osteoblast with APTG-CM induction and exhibited stem cell characteristics similar to that of normal human osteoblast-like cells.

MicroRNAs (miRNAs) are an integral part of this regulatory network with essential roles in pluripotent maintenance, proliferation and differentiation. The role of miRNA in osteogenic differentiation of MSCs has been indicated by several studies [19, 20]. However, few studies investigate the mechanism of miRNA in osteoblast differentiation of PDLSCs. It has been reported that ibandronate promote the proliferation of PDLSCs and enhance the expression of alkaline phosphatase (ALP), type I collagen (COL-1), osteoprotegerin (OPG), osteocalcin (OCN), and Runx2. The expression of miRNAs, including miR-18a, miR-133a, miR-141 and miR-19a, was significantly altered in the PDLSCs cultured with ibandronate [21]. Some results indicate that the 3D granules, in contact with hPDLSCs, showed not only osteoconductive properties, evaluated through the adhesion and proliferation process, but also the ability to stimulate VEGF secretion in hPDLSCs via miR-210 involvement. And miR- 2861, are involved in osteogenic differentiation and open a new scenario in the study of biomaterial performance [22, 23]. In this study, we applied miRNA sequencing to investigate the profile of miRNA expression in APTG-CM-induced and non-induced PDLSCs from 3 individual donors. Eleven miRNAs were significantly expressed in PDLSCs co-cultured with APTG-CM, and then miR-146a-5p was identified by computational miRNA target prediction analyses, which was up-regulated during miRNA expression profile. Using RT-qPCR, we validated the expression of miR-146a-5p, and the results of the expression level of miR-146a-5p were highly consistent with the results of miRNA sequencing. MiR-146a-5p differed from those highly expressed in human multipotent mesenchymal stromal cells (MSCs), such as hsa-miR-30c, hsa-miR-15b, and hsa-miR-130b, and the pattern of its expression in PDLSCs also differed from MSCs during osteogenic differentiation [24, 25]. Several previous studies have showed that miR-146a play a key regulatory role in MSC differentiation. They showed that miR-146a were significantly up-regulated in neuronally differentiated bone marrow-derived mesenchymal stem cells (BMSCs) [26]. Expression levels of miR-146a were dynamically changed during differentiation of hESCs to CD34^+^ hematopoietic cells, and in subsequent differentiation of the CD34^+^ cells into the erythroid lineage [27]. They also showed that miR-146a was up-regulated in differentiation of PDL cells with the help of ascorbic acid treatment and promoted the differentiation in PDL cells through the down-regulation of NF-kβ signaling [14]. The present study showed that the expression of miR-146a-5p was up-regulated in PDLSCs co-cultured with APTG-CM, and miRNA sequencing results were validated by RT-qPCR, which demonstrated that miR-146a-5p was involved in the promotion effect of APTG-CM on osteoblastic differentiation of PDLSCs. Our results suggested that miR-146a-5p might be a novel way in deciding the direction of PDLSCs differentiation.

## Conclusion

PDLSCs co-cultured with APTG-CM showed the osteoblastic differentiation of PDLSCs. MiRNA expression profiling and RT-qPCR results revealed that miR-146a-5p was up-regulated in APTG-CM-induced-PDLSCs compared with non-induced PDLSCs, which demonstrated that miR-146a-5p was involved in the promotion effect of APTG-CM on osteoblastic differentiation of PDLSCs. Our results suggested that miR-146a-5p might be a novel way in deciding the direction of PDLSCs differentiation.

## Data Availability

Data used in this study is available from the corresponding author upon reasonable request. The datasets generated and/or analysed during the current study are available in miRwork databases: (http://mirwalk.umm.uni-heidelberg.de/human/mirna/MIMAT0000449/) and TargetScan databases (https://www.targetscan.org/hsa-miR-146a-5p). Both mentioned weblinks above are direct persistent weblinks.

## Abbreviations

PDLSCs: Periodontal ligament stem cells
APTG-CM: Apical tooth germ cell-conditioned medium
HERS: Hartwig’s epithelial root sheath
MACS: Magnetic-activated cell sorting
